# Supplementary material for: Low Base-Substitution Mutation Rate in the Germline Genome of the Ciliate Tetrahymena thermophila
Source: Genome Biol Evol. 2016 Sep 15;8(12):3629–39. doi: 10.1093/gbe/evw223 (PMC5585995; doi:10.1093/gbe/evw223)
Supplement: Supplementary Data [file evw223_supp.zip › Supplementary_data_revised.pdf]

Supplement

July 28, 2016

1 Supplementary data file for GBE submission

The code and data underlying this analysis is available from https://github.com/dwinter/Tt_MA_supplement

In the course of analysing the *Tetrahymena* MA experiment we noticed a strange pattern.

A large number of apparent-mutations are shared by lines 47 and 51 (i.e. the same nucleotide substitution is present in both lines and absent from all others). Our "post processing" utility prints out read count matrices for such sites, here is a typical example of a "double mutant". Here each row represents the number of A,C,G and T bases mapped to this site in a given sample:

```
Skipping scf_8254582    675126
        A       C       G       T
TtM0    0       78      0       0
TtM19   0       59      0       0
TtM20   0       30      0       0
TtM28   1       80      0       0
TtM25   2       44      0       0
TtM29   0       30      0       0
TtM40   0       19      0       0
TtM44   0       170     0       0
TtM47   46      18      0       0
TtM50   0       172     0       0
TtM51   42      0       0       0
TtM531  0       83      0       0
```

This is typical in that one sample (47) has a minor portion of reads matching the ancestral (M0) allele while the rest match the apparent-mutant in 47. This document records and exploratory analysis of our data that investigates these two lines in particular.

1.1 Setup

The next cell clears the `namespace` of this notebook, and imports all the libraries needed to perform the analyses below.

```
In [1]: %reset -f

        import itertools
        from collections import defaultdict
        from collections import Counter

        import vcf

        from scipy import cluster
        from scipy import spatial
```

```
import pandas as pd

import Bio
from Bio import SeqIO
%pylab inline

print "_______Versions_______"
print "pyvcf:",vcf.VERSION
print "pandas:", pd.__version__
print "biopython:", Bio.__version__
! bedtools --version

#Make plots a bit bigger than the default
import matplotlib.pylab as pylab
pylab.rcParams['figure.figsize'] = 8, 5
```

```
Loading MoreUtils
Populating the interactive namespace from numpy and matplotlib
_______Versions_______
pyvcf: 0.6.8
pandas: 0.17.1
biopython: 1.66
bedtools v2.18.2-56-gb5e5070
```

1.2 Lines 47 and 51 have similar derrived allele frequencies

We first want to test of the similarity of 47 and 51 to each other (and difference from all other samples) extends beyond the small number of sites with putative mutations. To do this, we called variants from the experimental data using `gatk UnifiedGenotyper`. To properly capture the "mixed" genotypes described for line 47 above, we treated each sample as diploid and didn't filter any putative variants out:

```
gatk -R [path to reference] -T UnifiedGenotyper \
     -I [path to bam] #reads are [SRA IDS]      \
     -o desc_diploid_calls.vcf --sample_ploidy 2 -nct 4 -nt 8
```

This procedure calls all putative variants from the experimental data. Thus, the resultant `vcf` will have potentially heterozygous sites from the ancestral strain and artifacts produced from misalignment of reads associated with paralogy or copy number variation. Thus there will be many more apparent-variants produced than real mutations, allowing us to examine these patterns an a much broader scale. There are more than 80 000 such sites in our experiment:

```
In [2]: #loaded as list as we will iterate over these many times
        snps = [r for r in vcf.Reader(filename="data/desc_diploid_calls.vcf")]
        len(snps)
```

```
Out[2]: 85146
```

We are looking among-sample patterns in the reads mapped to each site. A straightforward way to investigate this is to extract the frequency of non-reference ("derived") alleles at each of the variant sites. Such derived alleles can represent real mutations, fixed (or heterozygous) differences between our ancestral strain and the reference genome or aberrant reads mapped to the genome.

Here we define functions to extract this information from a `vcf` record and generate a `numpy` array with one row for each site and one column for each sample:

```
In [3]: def get_sample_daf(s):
            if s.data.AD != None:
```

```
        return sum(s.data.AD[1:])/float(s.data.DP)
    return 0.0

def get_daf(rec):
    return np.fromiter( (get_sample_daf(s) for s in rec.samples), np.float64 )

daf_matrix = np.array([get_daf(r) for r in snps])
```

Having collected the derived allele frequency data, we want to see if some samples share similar patterns. Perhaps the simplest way to do this is cluster samples based on their allele frequency. `scipy` provides functions to create pairwise-distance matrices (based on Euclidean distance between the vectors of allele frequencies for each sample) and generate a clustering (with UPGMA):

```
In [4]: sample_names = [s.sample for s in snps[0].samples]
        dm = spatial.distance.pdist(np.transpose(daf_matrix))
        tree = cluster.hierarchy.dendrogram( cluster.hierarchy.linkage(dm),
                                             labels=sample_names,
                                             leaf_rotation =45)
```

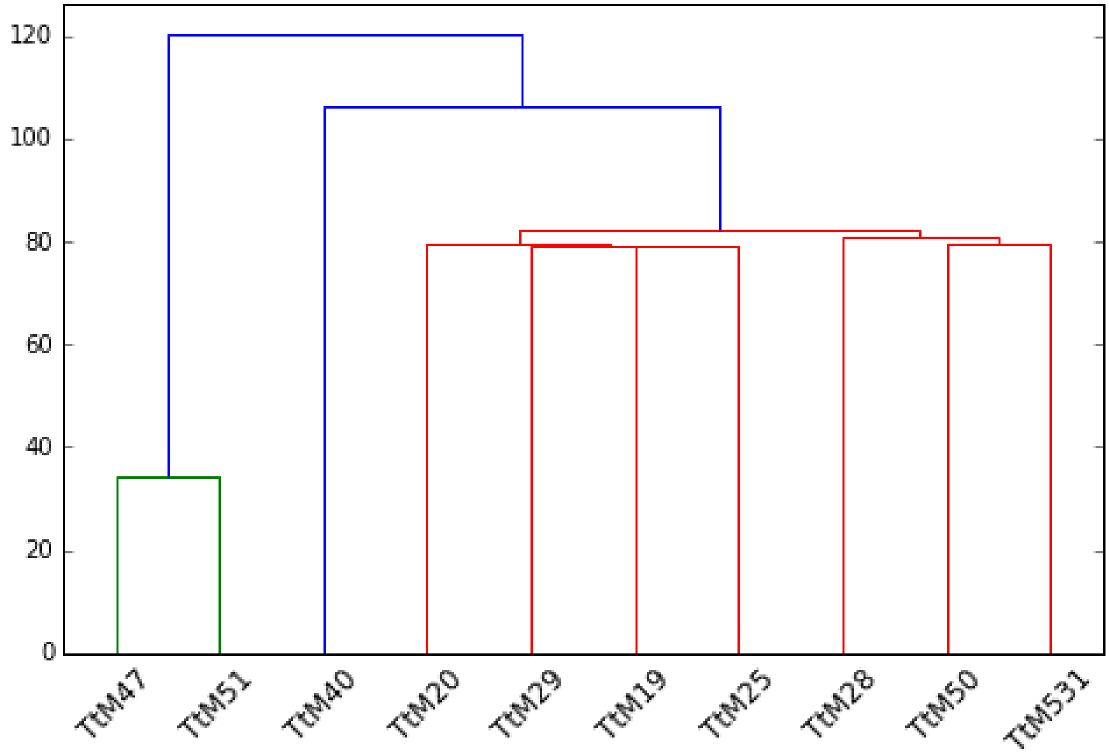

There is clearly something very different about lines 47 and 51, which much more similar too each other than any other two-sample pairing, and form a distinct cluster considerably distant from all others. Both of these observations suggest something systematic has happened to these two lines to make them different from the others.

1.3 There are many sites where only 47 and 51 lack derived alleles . . .

Much of the difference described above comes about because there are a large number of sites where only lines 47 and 51 *lack* derived alleles while all other samples have at least one read containing a derived allele. Here is the total number of sites with this pattern:

```
In [5]: mask_47_51 = np.array([x in ["TtM47", "TtM51"] for x in sample_names])
        sum( [(np.all(x[~mask_47_51]) > 0 and np.sum(x[ mask_47_51]) == 0) for x in daf_matrix] )
```

```
Out[5]: 11559
```

For the most part, these sites are not the result of all other lines being "fixed" for some non-reference allele. Rather the majority of sites have a mixture of reference and non-reference alleles at these. We can visualize this by created a stacked bar chart that describes the frequency of derived alleles across sites:

```
In [6]: def daf_specturm_plot(daf_mat, row_mask=None,sample_names=sample_names):
            """ """
            ramp = ['#3182bd', '#6baed6','#bdd7e7','#eff3ff'] #colourbrewer Blue5
            cuts = [1e-9, 0.25, 0.5, 0.75,  1.0]
            unmasked = np.transpose(daf_matrix[np.array(m)])
            bins = np.array([n for n,edge in [np.histogram(x,bins=cuts) for x in unmasked]]).transpose(
            start = np.sum(daf_matrix[np.array(m)] == 0,0)
            f = figure()
            ax = f.add_subplot(111)
            p = bar(np.arange(10), start, color='#08519c',)
            legends = [p[0]]
            for i,c in enumerate(bins):
                p =  bar(np.arange(10), c, color=ramp[i], bottom=start)
                start += c
                legends.append(p[0])

            legend(legends,
                   ["0", "(0-0.25)", "[0.25-0.5)", "[0.5.0.75)", "[0.75-1]"],
                   bbox_to_anchor=(1.4, 0.7),
               title= "Derrived allele frequency")

            ax.set_xticks(np.arange(10))
            ax.set_xticklabels(sample_names, rotation=45)

            show()
```

```
In [7]: m = np.array([(np.all(x[~mask_47_51]) > 0 and np.sum(x[mask_47_51]) == 0) for x in daf_matrix])
        daf_specturm_plot(daf_matrix, m)
```

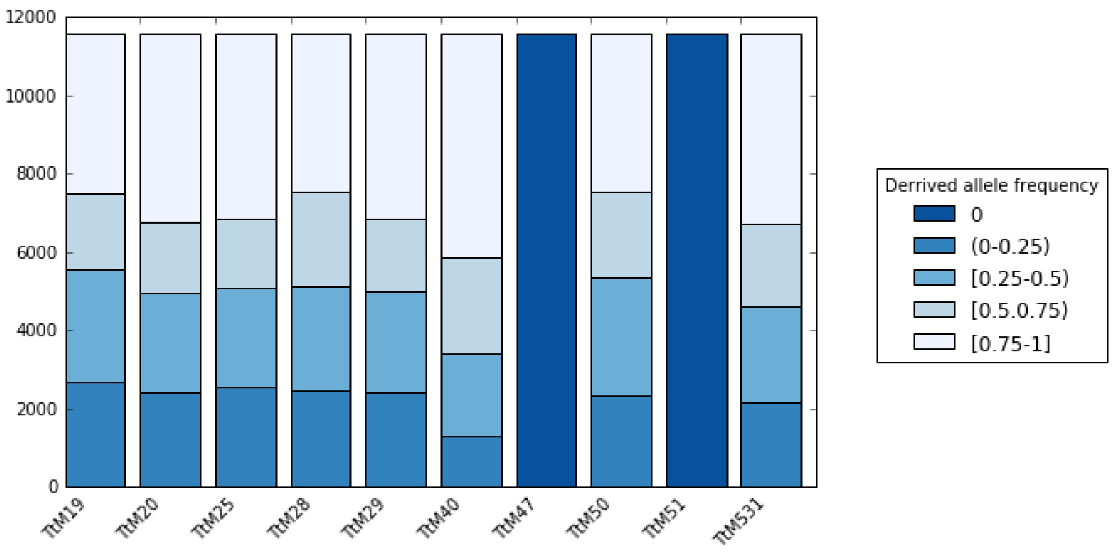

##...and many other sites where only 47 and 51 *have* derived alleles

Al tough there are not quite as many, there are also a large number of sites that have the opposite pattern: lines 47 and 51 have derived alleles and all other lines are "clean"

```
In [8]: m = [(np.all(x[mask_47_51]) > 0 and np.sum(x[~mask_47_51]) == 0) for x in daf_matrix]
        sum(m)
```

```
Out[8]: 1064
```

Again, most of these sites are not 'fixed' for derived alleles in lines 47 and 51. Rather, they mostly have intermediate frequencies of derrived alleles:

```
In [9]: daf_specturm_plot(daf_matrix, m)
```

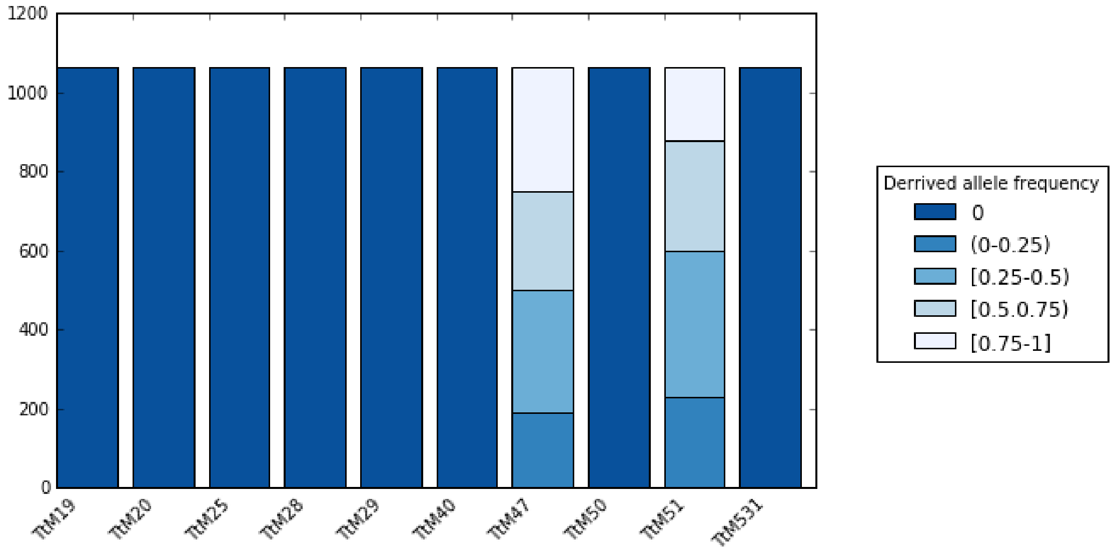

1.4 There many more sites that seperate 47 and 51 from all others than any other combination

We started investigating lines 47 and 51 because we already suspected something was strange about the data from these lines. To see how unusual it is that lines 47 and 51 share these patterns of missing (or uniquely present) derived alleles we can cacluate the number of sites showing such a pattern for all 2-sample combinations in our dataset:

```
In [10]: def different_duo(x, sample_names=sample_names):
             """
             For an array of allele frequences, if two and only two samples have frequencies of 0.0
             or if two and only two have frequences > 0 return the names of those samples
             x = array of frequences
             sample_names = list with each element being the name of a sample
             """
             is_zero = x==0.0
             if (sum(is_zero)==2):
```

```
            return "_".join([sample_names[i] for i,b in enumerate(is_zero) if b])
        if (sum(is_zero)==8):
            return "_".join([sample_names[i] for i,b in enumerate(is_zero) if not b])
        return None

    duo_count = Counter([different_duo(row) for row in daf_matrix if different_duo(row)])
```

```
In [11]: fig, ax = subplots()
         boxplot(duo_count.values())
         ax.annotate("47-51",
                     xy=(1,duo_count["TtM47_TtM51"]),
                     xytext=(1,duo_count["TtM47_TtM51"]-1000))
         ax.annotate("All other sample-combinations", xy=(1,1000), xytext=(1,1000))
         show()
```

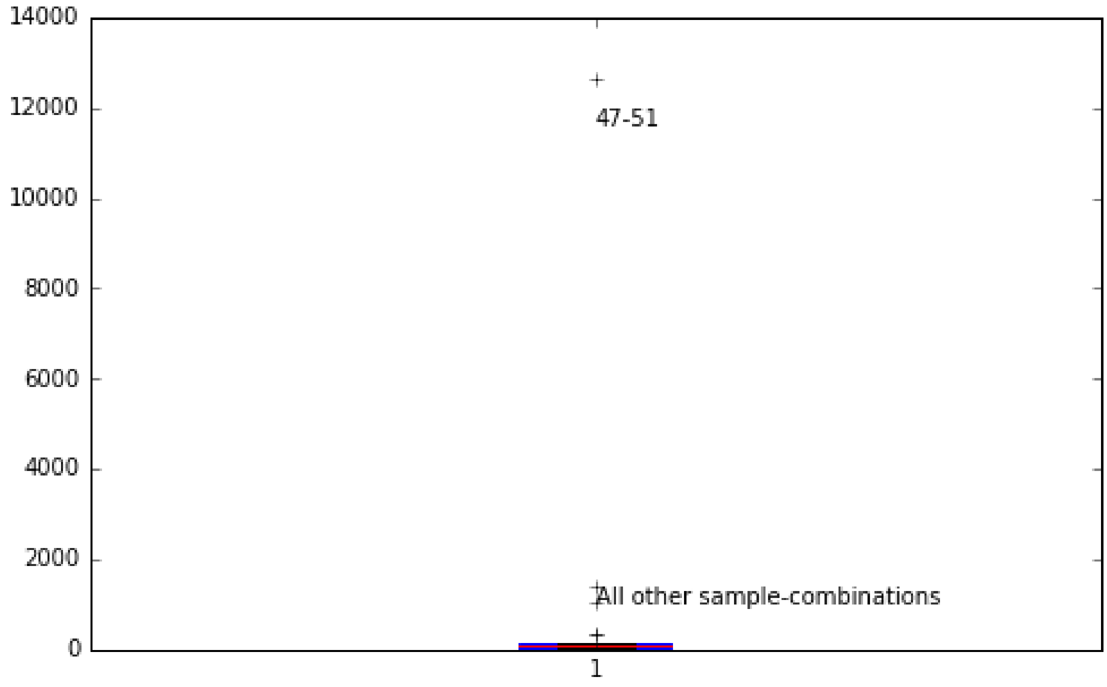

Clearly lines 47 and 51 represent extereme outliers.

1.5 Sites that seperate 47 and 51 occur all over the genome

So far, we have found that lines 47 and 51 differ from all samples in many more sites than any other pair of samples. To look further into this pattern we can see how sites in each of the classes we identified above (those for which 47 and 51 are unique in having derived alleles, and those ones where these samples are unique for not having derived alleles) are distributed across the genome. If this pattern is restricted to one or of sites it may represent an event whcih occured in one or a few chromosomes. On the other hand, if the pattern effects sites across the genome then something more systematic may be at play.

The reference genome for *T. thermophila* is still unfinished, and contains many short contigs. Thus, a plot of "site density" that required a statistic like 'sites per 10kb' might not be that helpful. Rather, we can plot the rank-order of sites in each class against their position in a concatenated genome. If sites were evenly distributed across the genome we'd expect to get a straight line, if they are restricted to a few regions it will be much more jagged.

In [12]:
```
#First identify sites matching each pattern
def derrived_alleles_unique(rec, mask):
    daf = get_daf(rec)
    if np.all(daf[mask] > 0):
        return sum(daf[~mask]) == 0
    return False

l47_51_only = [ r for r in snps if derrived_alleles_unique(r, mask_47_51)]
others_only = [ r for r in snps if derrived_alleles_unique(r, ~mask_47_51)]
```

In [15]:
```
#Now create a dictionary of chormosomes lengths from the reference genome
total_len = 0
seq_dict = {}
for rec in SeqIO.parse("data/tt-ref.fasta", "fasta"):
    seq_len = len(rec)
    seq_dict[rec.id] = (total_len, seq_len)
    total_len += seq_len
```

In [16]:
```
fig, ax = subplots(figsize=(8,7))
ylabel("Position across genome")
xlabel("Rank order of mutation")

fig.add_subplot(211)
plot([seq_dict[r.CHROM][0] + r.POS for r in l47_51_only])
title("Unique to 47 and 51")
axhline(total_len, color="grey")

fig.add_subplot(212)
plot([seq_dict[r.CHROM][0] + r.POS for r in others_only ])
title("Shared by all samples other than 47 and 51")
axhline(total_len, color="grey")
fig.tight_layout()
show()
```

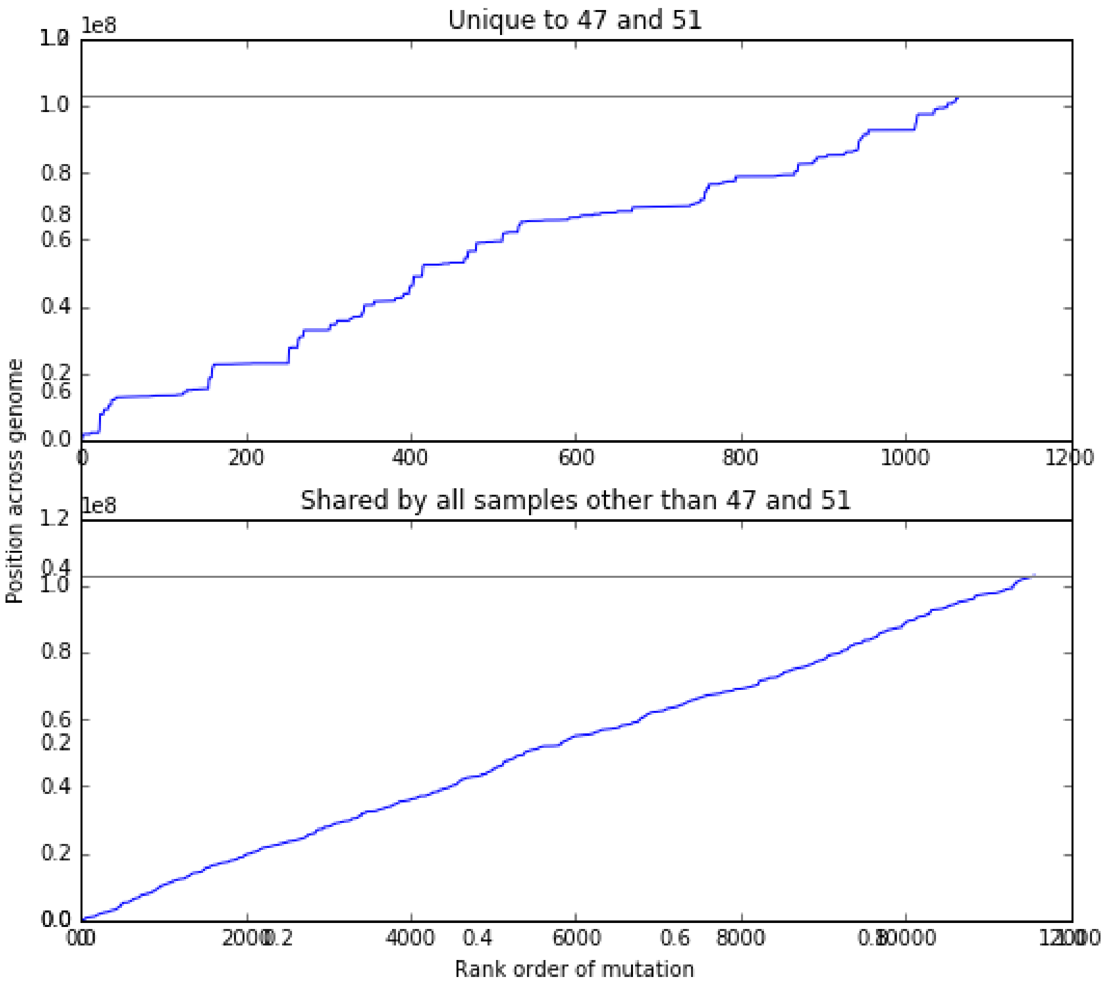

Sites where all samples other than 47 and 51 have at least one derrived allele are well distributed across the genome. Sites that have derived alleles in 47 and 51 only are a little more patchily distributed: there seem to be some long streches with no sites. It should be noted however, there are much fewer sites in this class.

We can also see how many contigs have at least one site in each class, and what proportion of the reference genome is contained in affected contigs:

```
In [17]: def effected_chroms(sites, genome_size=total_len):
             included = set([(r.CHROM, seq_dict[r.CHROM][1]) for r in sites])
             n = len(included )
             p = sum([j for i,j in included])/float(total_len)
             print "{0} contigs contaning {1:.2f} of the genome".format(n,p)

         effected_chroms(l47_51_only)
```

```
125 contigs contaning 0.26 of the genome
```

```
In [18]: effected_chroms(others_only)
```

```
479 contigs contaning 0.97 of the genome
```

1.6 Sites that separate 47 and 51 occur in 'clumps'

The patterns that separate these two lines are 'global' in the sense they occur all over the genome. We can also looker at their distribution at a finer scale and ask if they "clump together". That is, are sites that fall into one of these classes likely to be close to another site of the same class (or alternatively to sites from the other class).

To look at how close sites matching either pattern are to each other we will use `bedtools`, which is a command line program that takes interval (bed, vcf, gff...) files as input. so we need to first write out new vcf files for each "class" of sites:

```
In [19]: def write_vcf(recs, fname, template=vcf.Reader(filename="data/desc_diploid_calls.vcf")):
             with(open(fname, "w")) as out:
                 vcf_out = vcf.Writer(out,template )
                 counter = 0
                 for r in recs:
                     vcf_out.write_record(r)
                     counter += 1
             return counter

         print "Wrote", write_vcf(l47_51_only ,"unique_4751.vcf"), "recs"
         print "Wrote", write_vcf(others_only ,"not_4751.vcf"), "recs"
```

```
Wrote 1064 recs
Wrote 11559 recs
```

`bedtools` can then calculate the inter-site distances. Since it will add these distances as the last column of the outputted files we will use `cut` to store just these numbers. (Sites with no neighbouring sites on the same contig will be returned as -1, we will just skip over these sites below):

```
In [20]: d_unique = ! bedtools closest -d -io -a unique_4751.vcf -b unique_4751.vcf | cut -f 39
         d_others = ! bedtools closest -d -io -a not_4751.vcf -b not_4751.vcf | cut -f 39
         d_between = ! bedtools closest -d  -a not_4751.vcf -b unique_4751.vcf | cut -f 39
         d_summary = np.array(
          [ np.fromiter( (int(i) for i in x if i), np.int32) for x in [d_unique, d_others, d_between] ]
         )
```

As a result of the above commands `d_summary` is now a list with three elements. Those elements are `numpy` arrays, the first two containing the smallest inter-site distances *within* the two class of "47 51 unique" sites, and the third containing the same for sites *between* classes.

We can now compare the distribution of these distances

```
In [21]: figure()
         boxplot(d_summary)
         show()
```

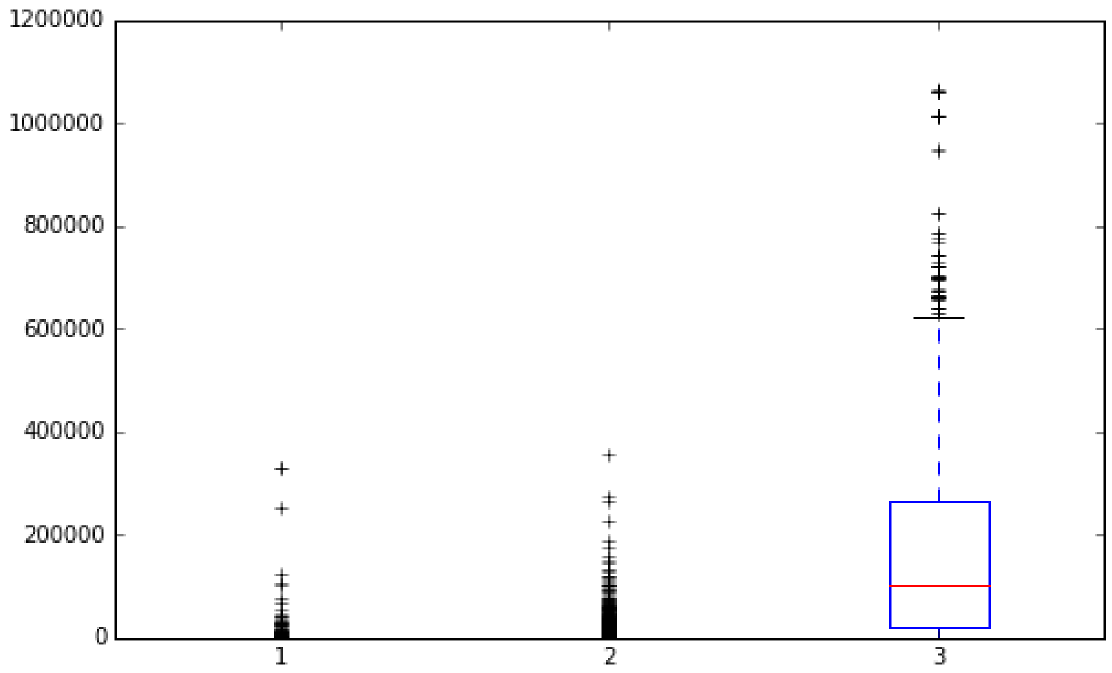

Clearly, the sites that fit into one of these classes are likely to be close to another one. That same pattern doesn't apply to sites in different classes

The median distance between sites of the same class is tiny (2 or 3) and 80–90% of sites in a given class are within 50 bases of another site in the same class. Given our sequencing reads are 100 base pairs long it seems like these derived-alleles are on the same DNA molecule:

```
In [22]: [median(x) for x in d_summary]

Out[22]: [3.0, 2.0, 100269.0]

In [23]: [round(mean(x <= 50),3) for x in d_summary]

Out[23]: [0.833, 0.877, 0.036]
```

1.7 Sites with derrived alleles in all samples other than 47 and 51 generally have low sequencing coverage

Taken together, these results are starting to suggest a mis-mapping of reads is contributing to the difference between 47 and 51. Sequencing coverage also contains information about mapping, so to investigate this pattern further we can look at coverage data from sites in each of the classes discussed above.

`grand_cov_summary.csv` is a per-contig, per-sample summary of all the experimental data, which we generated during QA of this project. The important columns for here are `sample`, `chrom` and `median coverage`. From these data we can determine how much the coverage at a site differs from the normal level, while accounting for differences among samples and contigs:

```
In [24]: covs = pd.DataFrame.from_csv("data/grand_cov_summary.csv")
         covs.head()

Out[24]:                len  n_mappable sample  median_coverage   variance  p.uncovered  \
         chrom
         scf_8253803   8327        1238    TtM0                0  380.85170     0.675945
```

```
scf_8253803  8327        1238  TtM51                0  104.93690     0.568980
scf_8253803  8327        1238  TtM29                0   55.87656     0.644706
scf_8253803  8327        1238  TtM19                0  129.43970     0.571047
scf_8253803  8327        1238  TtM50                0  505.90310     0.755439

             n.telomeres  mean_cov high_cov  weird
chrom
scf_8253803            0         0    False  False
scf_8253803            0         0    False   True
scf_8253803            0         0    False  False
scf_8253803            0         0    False  False
scf_8253803            0         0    False  False
```

We can use this data to create a (nested) dictionary that allows us to retreive the median coverage of each contig in the reference genome for every sample:

```
In [25]: coverage_dictionary = defaultdict(lambda: defaultdict(dict))
         for chrom, details in covs.iterrows():
             coverage_dictionary[ details["sample"] ][ chrom ] = details["median_coverage"]

         coverage_dictionary["TtM50"]["scf_8253803"]
```

```
Out[25]: 0.0
```

... and we can use that data to compare the coverage at a given site to the "normal" coverage for that sample in that contig. Specifically, we can generate log2 fold differences so that +1.0 is a doubling of the normal overage and -1.0 is a halving:

```
In [26]: def cov_fold_diff(s, median, eps=0.01):
             if s.data.DP:
                 if median > 0:
                     return  np.log2(float(s.data.DP)/ median)
                 else:
                     #return np.log2(float(s.data.DP)/eps)
                     return np.nan

             if median > 0:
                 #return np.log2(eps/median)
                 return np.nan
             return 0.0

         def extract_cov_folds(r, cov_dict = coverage_dictionary):
             return np.array([cov_fold_diff(s, cov_dict[s.sample][r.CHROM]) for s in r.samples ])
```

Finally we can compare the distribution of coverage differnces across sites:

```
In [27]: def mask_nans(arr):
             """
             Turn a 2D array including NaNs values into a list of lists, with each
             one with alll of the non-NaN for a sub-array.

             matplotlib boxplot no longer accepts NaNs, Nones or masked arrays.
             In order to produce a boxplot, we need to extract only those values
             that are not NaNs for each sample. In turn, that means each sample
             will have a different number of valid observations. This means the values
```

```
    can no longer be store in arrays (where each element must have the same
    dimension). Taken togetheher, this means converting the arrays of coverage
    differences into a list of lists.
    """
    return [[obs for obs in row if obs] for row in ma.masked_invalid(arr.T)]


cov_diff_u = np.array([extract_cov_folds(r) for r in l47_51_only])
cov_diff_o = np.array([extract_cov_folds(r) for r in others_only])

cv = ["#9ecae1" if s in ["TtM47", "TtM51"] else "#084594" for s in sample_names]

fig, ax = subplots(figsize=(12,5))
ax.xaxis.set_visible(False)
ax.yaxis.set_visible(False)

fig.add_subplot(121)
title("Sites where only 47 and 51 have derived alleles")
bplot = boxplot(mask_nans(cov_diff_u), patch_artist=True)
for patch, col in zip(bplot['boxes'], cv):
    patch.set_facecolor(col)

fig.add_subplot(122)
title("Sites where all sites other than 47 and 51 have derived alleles")
bplot = boxplot(mask_nans(cov_diff_o), patch_artist=True)
for patch, col in zip(bplot['boxes'], cv):
    patch.set_facecolor(col)
fig.tight_layout()
show()
```

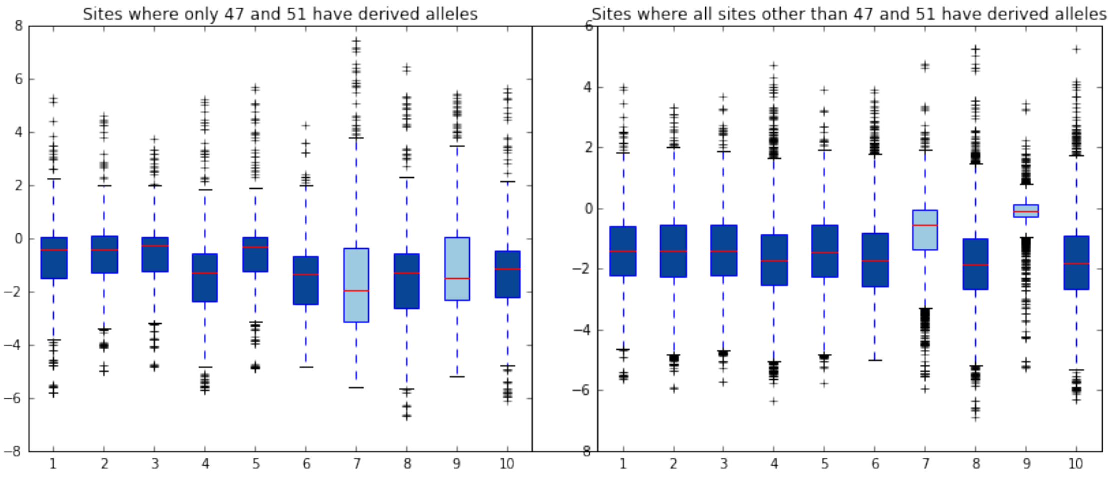

The second plot is particularly striking - sites at which lines other than 47 and 51 always have derived alleles generally have lower coverage. All samples have a large number of outliers, suggested these sites may also include copy-number variants or paralagous sequences that produce mapping errors.

2 Conclusion

It is overwhelming clear that lines 47 and 51 systematically differ from all others . These two samples have a consistent pattern of derived-allele frequencies that makes them different than all other samples. There are thousands of sites at which these samples are either (a) unique for containing derived alleles or (b) unique for lacking derived alleles.Sites matching these patterns occur all over the genome, but fall into distinct clusters. Finally, it seems sites for which every sample other than 47 and 51 have derived alleles have unusually low sequencing coverage.

Why these two samples differ from others is less clear. It is possible that the sequencing libraries produced from these two lines, and noy others, contain a high proportion of usually MIC-limited sequences which in turn has led to systematic differences in the way reads have been mapped to the reference genome. A test of this or other hypotheses that might explain these patterns is beyond the scope of the present work.

Whatever the cause of this pattern, it's clear that these samples are systematically different from others. Because we cannot say precisely what process has generated this difference it is not possible to control for it, or incorporate this process into our mutation calling model. Moreover, it is impossible for us to tell whether putative mutations in these lines are indeed true mutations accrued during our MA experiment, or simply products of the same aberration that has influenced data at other sites. For this reason we exclude these lines in all of mutation rate estimates.
